# Successful Heart Transplantation After 7 h of Cold Storage and Paracorporeal Donor Heart Resuscitation

**DOI:** 10.3389/ti.2022.10740

**Published:** 2022-10-10

**Authors:** Rymbay Kaliyev, Timur Lesbekov, Makhabbat Bekbossynova, Zhuldyz Nurmykhametova, Assel Medressova, Aidyn Kuanyshbek, Linar Faizov, Robertas Samalavicius, Yuriy Pya

**Affiliations:** ^1^ National Research Center for Cardiac Surgery, Nur-Sultan, Kazakhstan; ^2^ Vilnius University Hospital Santaros Klinikos, Vilnius, Lithuania

**Keywords:** heart transplantation, donor heart recuscitation, cold storage, *ex vivo* perfusion, cytokine adsorption

Dear Editors,

The need of organs for transplantation is increasing worldwide, though the availability of them does not meet the existing demand (1). In some countries, factors limiting even “ideal” heart utilization include the distance between the donor and recipient hospitals, weather conditions (2). “Suboptimal” organs are more often used for transplantation (3). The risk of primary graft failure and death rises dramatically for the heart as ischemic time increases (4). Real life practice demonstrates that accidental rescue might become an acceptable strategy in the future.

We report a case of paracorporeal donor heart resuscitation followed by successful heart transplantation after 7 h of cold storage.

Fifty-five years old male patient was diagnosed with dilated cardiomyopathy at 2012 years. He underwent a HeartMate3 left ventricular assist device (LVAD; Abbott Inc.) implantation as a bridge to heart transplantation (HTx) in January 2018. His clinical status was complicated by a deep driveline infection. After initial antibiotic therapy, the patient underwent two driveline site debridement surgeries in May and September 2019. Recurrent infections with resistant pathogens (*Burkholderia cepacia*, *Staphylococcus aureus*) resulted in a higher waitlist status priority for HTx.

A suitable donor heart became available from a 42-year-old male who had had a hemorrhagic stroke. Before explant for hemodynamic stabilization, he needed infusion of norepinephrine 0.1 mcg/kg/min and dobutamine, 15 mcg/kg/min. Echocardiographic evaluation of the donor heart showed (LVEF 62%) no abnormalities, 2 days in ICU and 1 day on ventilator. Donor and recipient had the blood group O (I) Rh-positive and AB (IV) Rh-positive status, respectively. Our institution is sole transplant center in the country, and donor hearts are often retrieved from distant regions to be transplanted (5). This time the distance between the two hospitals was 1,000 km. Furthermore, poor winter weather conditions delayed the flight of organ retrieval team. Therefore, travel time on a return journey was estimated to be at least 6 h. The donor heart was arrested with the standard heart preservation solution (4°C Custodiol) and preserved in standard way of cold ischemic storage.

When the donor heart arrived to the clinic, the time of cold ischemic storage was already 430 min. Severe, time consuming, and technically demanding reoperation for LVAD explantation anticipated additional time. Due to the high risk of graft failure and post implantation dysfunction, the decision was made to perfuse the allograft through cardiopulmonary bypass (CPB) circuit of recipient and assess its function after reperfusion. The ascending aorta of donor heart was connected in end-to-end manner to splitted systemic flow line *via* 3/8 connector. No vents were used making open blood drainage from organ chambers into container and then to cardiotomy reservoir through suction pump. Standard gases and lactate samples of freely draining blood were collected from the container and measured during paracorporeal resuscitation stage.

Following 6 min of reperfusion of the heart, stable sinus rhythm was restored, using the 15 J shock from external biphasic defibrillator. The standard CPB roller pump was used (Stockert S5, LivaNova Deutschland GmbH), with the mean aortic pressure between 68 and 84 mmHg and coronary blood flow between 800–860 ml/min at a temperature of 35C. The graft was conditioned with 45 μg/kg Levosimendan infusion in the coronary perfusion and cytokine adsorption (CytoSorb, Cytosorbents Inc., NJ, United States) with a blood flow of 200–300 ml/min was applied from recipient’s CPB circuit. Periodic arterial and venous blood samples were taken from donor heart during perfusion period of 54 min. After organ evaluation, the decision was made to use the heart for transplantation based on surgeon observation, good visual contractility, stable sinus rhythm, and lactate values. The donor heart was arrested with 1 L of regular normothermic blood cardioplegia solution just before transplanting. It took two cardioplegia doses before resuming systemic blood flow in the heart. The bi-atrial technique of performing orthotopic cardiac transplantation has been utilized. In 52 min, the heart rhythm was restored. Transplantation and preoperative care proceeded according to the standard procedures of our center. The next day after HTx the remnants of driveline and ICD with leads were extracted. Prolonged suppressive antibiotic therapy with Colistin, Meropenem and Vancomycin in combination with Cansidas was used up to 4 weeks.

Total cold ischemic time was 430 min and duration of coronary perfusion was 54 min. This resulted in a total cross-clamp to cross-clamp time of 534 min (Figure 1). Venous lactate taken from freely draining blood were collected from the donor heart container at the start of coronary perfusion was 2.2 mmol/L, and 3.0 mmol/L at the end. Total CPB time of the recipient was 183 min including 40 min of warm reperfusion after transplantation. Patient was weaned from cardiopulmonary bypass administrating norepinephrine 0.1 mcg/kg/min and dobutamine 7 mcg/kg/min infusion. The recipient was extubated 23 h following surgery, discharged from the hospital 10 days after. The heart function was measured by Swan-Ganz catheter showing 6.4 L/min of cardiac output and 4 L/min/m^2^of cardiac index. Echocardiography parameters confirmed normal biventricular function: S^1^LV lateral 21 cm/s, S^1^LV medial 7.8 cm/s, S^1^RV 7.6 cm/s, LVEF 68%. For over 9 mon post-transplant, there is no evidence of rejection or cardiac dysfunction.

**FIGURE 1:**
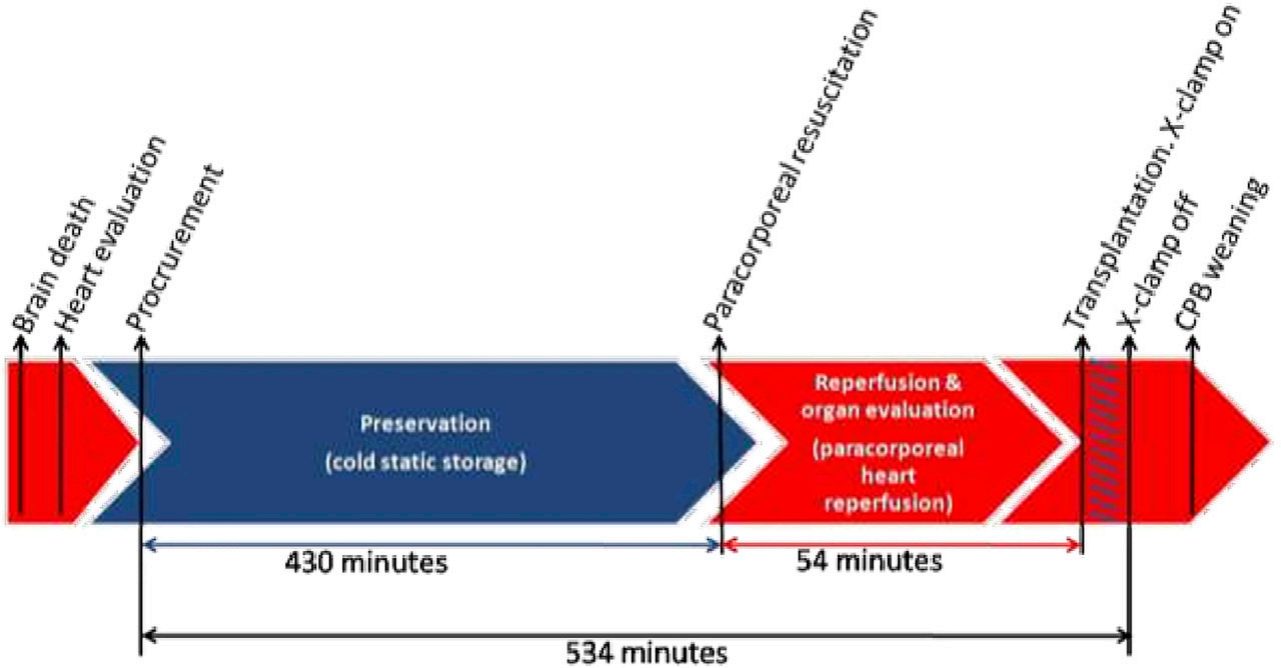
Process of heart transplantation.

With prolonged ischemic time, the donor graft can be transported for longer distances and increase the chances of gaining a matching donor graft. We report a successful heart transplantation following donor heart resuscitation after 7 h of cold storage using recipient’s cardiopulmonary bypass circuit prior to organ implantation. This maneuver allowed to start resuscitation of the donor organ by means of controlled warm reperfusion, medical treatment and cytokines adsorption. It is remarkable that this scenario was covered by recipient’s physiologic conditions, i.e., patient’s multiorgan system reserve capacity.

To the best of our knowledge, this is the first reported case of paracorporeal resuscitation of donor heart after 7 h of cold static storage using recipient’s cardiopulmonary bypass circuit to be successfully transplanted.

## Data Availability

The original contributions presented in the study are included in the article/supplementary material, further inquiries can be directed to the corresponding author.

## Abbreviations

ICU, intensive care unit; LVAD, left ventricular assisted device; CPB, cardiopulmonary bypass; LVEF, left ventricular ejection fraction; S1LV, myocardial velocity associated with isovolumic contraction of left ventricle; S1RV, myocardial velocity associated with isovolumic contraction of right ventricle.
